# Enhancing poly(3-hydroxyalkanoate) production in *Escherichia coli* by the removal of the regulatory gene *arcA*

**DOI:** 10.1186/s13568-016-0291-z

**Published:** 2016-11-23

**Authors:** Ryan A. Scheel, Liyuan Ji, Benjamin R. Lundgren, Christopher T. Nomura

**Affiliations:** 1 Department of Chemistry, State University of New York College of Environmental Science and Forestry, 1 Forestry Drive, Syracuse, NY 13210 USA; 2 Center for Applied Microbiology, State University of New York College of Environmental Science and Forestry, 1 Forestry Drive, Syracuse, NY 13210 USA; 3 Hubei Collaborative Center for Green Transformation of Bio-Resources, College of Life Sciences, Hubei University, Wuhan, 430062 China

**Keywords:** Polyhydroxyalkanoates, Biodegradable polymer, *Escherichia coli*, Fatty acid metabolism, *arcA*, Beta-oxidation

## Abstract

Recombinant *Escherichia coli* is a desirable platform for the production of many biological compounds including poly(3-hydroxyalkanoates), a class of naturally occurring biodegradable polyesters with promising biomedical and material applications. Although the controlled production of desirable polymers is possible with the utilization of fatty acid feedstocks, a central challenge to this biosynthetic route is the improvement of the relatively low polymer yield, a necessary factor of decreasing the production costs. In this study we sought to address this challenge by deleting *arcA* and *ompR,* two global regulators with the capacity to inhibit the uptake and activation of exogenous fatty acids. We found that polymer yields in a *ΔarcA* mutant increased significantly with respect to the parental strain. In the parental strain, PHV yields were very low but improved 64-fold in the *ΔarcA* mutant (1.92–124 mg L^−1^) The *ΔarcA* mutant also allowed for modest increases in some medium chain length polymer yields, while weight average molecular weights improved by approximately 1.5-fold to 12-fold depending on the fatty acid substrate utilized. These results were supported by an analysis of differential gene expression, which showed that the key genes (*fadD, fadL,* and *fadE*) encoding fatty acid degradation enzymes were all upregulated by 2-, 10-, and 31-fold in an Δ*arcA* mutant, respectively. Additionally, the short chain length fatty acid uptake genes *atoA, atoE* and *atoD* were upregulated by 103-, 119-, and 303-fold respectively, though these values are somewhat inflated due to low expression in the parental strain. Overall, this study demonstrates that *arcA* is an important target to improve PHA production from fatty acids.

**Electronic supplementary material:**

The online version of this article (doi:10.1186/s13568-016-0291-z) contains supplementary material, which is available to authorized users.

## Introduction

Poly(3-hydroxyalkanoates), or PHAs, are a group of biodegradable polyesters produced by a variety of microorganisms as a form of carbon storage (Lu et al. 2009). These PHAs are typically classified as short chain-length (SCL) PHAs, which contain repeating units of 3–5 carbons, and medium chain-length (MCL) PHAs containing 6–14 carbons. The physical properties of PHAs are dependent on monomer composition; SCL PHAs are generally stiff and brittle while MCL PHAs are more elastomeric, and co-polymerization of the two groups allows for great variability in material properties (Laycock et al. 2013). Previous studies have shown that MCL PHAs can be effectively produced in recombinant *E. coli* lacking the fatty acid degradation gene *fadB* when utilizing a related carbon source such as fatty acids, although the monomer composition of the resulting polymers was heterogenous and uncontrolled (Langenbach et al. 1997; Qi et al. 1997).

**Fig. 1:**
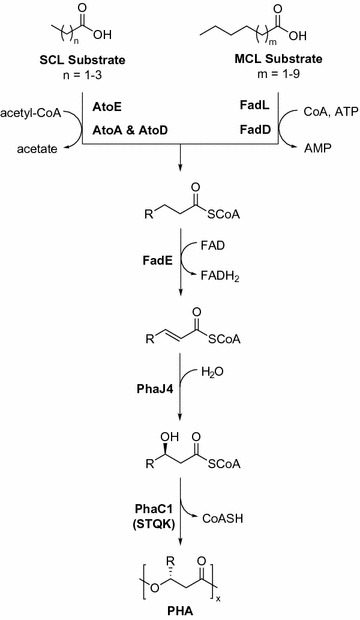
Biosynthesis of PHA in *E. coli* LSBJ utilizing short-chain-length (SCL) and medium-chain-length (MCL) fatty acids. The absence of *fadB* and *fadJ* in *E. coli* LSBJ in combination with the plasmid-borne recombinant enzymes PhaC1(STQK) and PhaJ4 establishes a linear pathway for the production of PHA polymers from free fatty acids. Extracellular fatty acids are transported across the outer membrane dependent on size; SCL and shorter MCL fatty acids can diffuse across the outer membrane, while longer MCL fatty acids can be transported by the long-chain fatty acid transporter FadL (Lepore et al. 2011). Inner membrane transport and activation is accomplished by the SCL-specific Ato system (AtoEAD) or the MCL-specific acyl-CoA synthetase FadD (Kameda and Nunn 1981; Theodorou et al. 2006). Acyl-CoA substrates are converted into enoyl-CoA by the acyl-CoA dehydrogenase enzyme FadE (Campbell and Cronan 2002), and are unable to proceed further through β-oxidation due to the absence of FadB and FadJ. The enoyl-CoA pool is then converted to (*R*)-3-hydroxyacyl-CoA by the *R*-specific enoyl-CoA hydratase PhaJ4 (Tsuge et al. 2003), and finally polymerized by the PHA synthase PhaC1(STQK) (Takase et al. 2003, 2004). This system allows for the biosynthesis of PHA polymers with tightly controlled repeating unit composition, as the number of carbons present in the fatty acid substrate is retained as the total number of carbons in each repeating unit

Recently, it was shown that the monomer identity can be precisely controlled in both PHA homo- and co-polymers synthesized by recombinant *Escherichia coli* strain LSBJ (Tappel et al. 2012a, b). This was accomplished by deleting both the *fadB* and *fadJ* genes in *E. coli* LS5218, recombinantly co-expressing the *phaJ4* gene from *Pseudomonas putida* KT2440 with the highly active and broad substrate utilizing *phaC1(STQK)* genes, and feeding in specific ratios of fatty acids for conversion to PHAs (Fig. 1). This system allowed for strict control of repeating unit composition which enables great control over the physical properties of PHA polymers produced using this system, unlocking the potential for tailoring PHA materials for click-chemistry modifications (Levine et al. 2015, 2016; Pinto et al. 2016). Although these previous studies addressed control of the monomer composition and thus physical and chemical properties of PHA polymers, overall polymer yields were still relatively low and some fatty acid substrates had poor incorporation into either PHA homo- or copolymers. The previously defined system relied heavily on the deletion of the *fadR* gene in *E. coli* LS5218 for constitutive expression of the genes encoding enzymes from the β-oxidation pathway (Spratt et al. 1981). In a previous study, researchers demonstrated that the inhibition of β-oxidation intermediates using acrylic acid was an effective strategy for improving PHA biosynthesis, particularly in combination with a *fadR* deletion (Qi et al. 1998). In addition to FadR, there are three other transcriptional regulators, ArcA, OmpR, and CRP-cAMP, that are known to inhibit the expression of genes involved in β-oxidation. For this study, we focused on the regulators ArcA and OmpR because CRP-cAMP is known to act as a transcriptional activator of β-oxidation in the absence of glucose, and only exhibits repression when glucose is present (Fic et al. 2009). We hypothesized that removal of transcriptional regulators that inhibit expression of β-oxidation related genes would result in improved flux through fatty acid catabolic pathways to increase PHA polymer yields in our engineered system. Therefore, in this study *E. coli* LSBJ was engineered to improve the biosynthesis of PHA from fatty acid substrates by removing the global regulatory genes *arcA* and *ompR*.

The transcriptional regulator OmpR functions as a response regulator of the two component regulatory EnvZ/OmpR system, which exhibits control over the expression of outer membrane porins in response to osmolarity (Mizuno and Mizushima 1990). The sensor kinase EnvZ autophosphorylates in response to extracellular osmolarity and transphosphorylates OmpR (OmpR-P), which binds to DNA and alters expression of genes within the OmpR regulon (Forst and Roberts 1994; Matsubara and Mizuno 1999). The most well-studied members of the OmpR regulon are OmpF and OmpC, outer membrane porins that control the diffusion of small hydrophilic molecules (Aiba and Mizuno 1990; Mizuno and Mizushima 1990; Silhavy and Pratt 1995). However, OmpR controls numerous other transporter genes including *fadL* (Table 1), as well as genes involved in amino acid metabolism and flagellar biosynthesis (Higashitani et al. 1993; Oshima et al. 2002).

**Table 1:** Regulation Targets of ArcA and OmpR

Target Gene	Description	Reference
*arcA*		
* fadL*	Long-chain fatty acid transporter, experimental evidence	Cho et al. (2006)
*fadD*	Acyl-CoA synthetase, experimental evidence	Cho et al. (2006)
* fadE*	Acyl-CoA dehydrogenase, experimental evidence	Cho et al. (2006)
*ompR*		
*fadL*	Long-chain fatty acid transporter, predicted	Higashitani et al. (1993)

The global transcriptional dual regulator ArcA functions as the response regulator of the two-component regulatory ArcAB system, which regulates the expression of genes involved in aerobic and anaerobic metabolism in response to oxygen availability (Iuchi and Ec 1988; Lynch and Lin 1996). During conditions of decreasing oxygen availability, ArcA is activated through a phosphorelay mechanism and binds to the consensus sequence 5′-wGTTAATTAw-3′ (w is A or T) located in numerous genes, including several genes involved in β-oxidation shown in Table 1, and acts as either a repressor or activator (Iuchi and Lin 1992; Lynch and Lin 1996; Liu and Wulf 2004; Cho et al. 2006; Peña-Sandoval and Georgellis 2010). It has previously been shown that the redox biochemistry and transcriptional regulation of an Δ*arcA* mutant strain is significantly altered during microaerobic growth conditions, and to a lesser degree during aerobic growth conditions (Oshima et al. 2002; Alexeeva et al. 2003; Shalel-Levanon et al. 2005). The work presented here demonstrates for the first time an *arcA* deletion mutant combined with a *fadR* mutation to improve the biosynthesis of PHA polymers derived from fatty acid substrates.

## Methods

### Materials

A complete list of strains, plasmids, and primers used for this study is shown in Table 2. All strains were grown in Lennox Broth (LB; composition per liter: 10 g tryptone, 5 g yeast extract, and 5 g sodium chloride, pH 7.0) purchased from Difco, and the antibiotics kanamycin (50 mg L^−1^) and ampicillin (100 mg L^−1^) were added to media throughout the experiment as appropriate. The fatty acids sodium butyrate (Alfa Aesar), pentanoic acid (Alfa Aesar), hexanoic acid (Alfa Aesar), heptanoic acid (Alfa Aesar), sodium octanoate (Sigma Aldrich), decanoic acid (Alfa Aesar), and dodecanoic acid (Acros Organics) were used as feed supplements for PHA production (12 mM), along with the surfactant Brij-35 (Fisher Scientific, 4.0 g L^−1^). Sodium phosphate dodecahydrate (Acros Organics, 8 mM) was added when noted. Sodium hydroxide (5 M) was used to adjust the pH to 7.0 when necessary. Primers were ordered from Integrated DNA Technologies (IDT). ACS HPLC-grade chloroform and methanol were used for gas chromatography (GC) sample preparation and polymer purification.

**Table 2:** Strains, plasmids, and primers

*Escherichia coli*	Relevant characteristics	Source or reference
LSBJ	*fadB::Cm,* Δ*fadJ, atoC512* (Const), *fadR601*	Tappel et al. (2012a)
RSC02	*ΔarcA* LSBJ	This study
RSC04	*ΔompR* LSBJ	This study
RSC06	*ΔarcA, ΔompR* LSBJ	This study
*Plasmids*		
pKD46	λ Red recombinase expression plasmid; expresses *exo, β,* and *γ* genes from λ phage; P-_araB_ promoter; *araC*; Amp^R^; temperature sensitive replicon	Datsenko and Wanner (2000)
pKD13	Neomycin phosphotransferase flanked by FLP recombinase recognition targets, Amp^R^, Km^R^	Datsenko and Wanner (2000)
pCP20	FLP recombinase expression plasmid, Amp^R^, temperature sensitive replicon	Datsenko and Wanner ( 2000)
pBBR-C1J4SII	pBBR1MCS-2 derivative Δ*phaAB*, *phaJ4, phaC1* (STQK)	Tappel et al. (2012b)
*Primers* ^a,b^	*Sequence (5′ to 3′)*	
pKD13.F.*arcA*	ATGCAGACCCCGCACATTCTTATCGTTGAAGACGAGTTGGTAACACGCAAGTGTAGGCTGGAGCTGCTTC	
pKD13.R.*arcA*	TTAATCTTCCAGATCACCGCAGAAGCGATAACCTTCACCGTGAATGGTGGATTCCGTGGATCCGTCGACC	
pKD13.F.*ompR*	ATGCAAGAGAACTACAAGATTCTGGTGGTCGATGACGACATGCGCCTGCGGTGTAGGCTGGAGCTGCTTC	
pKD13.R.*ompR*	TTAGAACATTACCTTATGACCGTACTGCTCAAGAATGCCTTTCACGCGTTATTCCGTGGATCCGTCGACC	
*arcA*.check.F/R	GTTAATTTGCAGCATGCATCAGG/GACGATGAGTTACGTATCTGG	
*ompR*.check.F/R	AAATTGTTGCGAACCTTTGG/GCAATAACGTACGGGCAAAT	
qAtoA.F/R	GGTGCAGCCATGTTTGATAG/CGCGAGGTTTGCTTCTTC	
qAtoD.F/R	ACTTGGCAACCTGACCTATC/GACCAGTTCATCTGGCTCTAC	
qAtoE.F/R	ACTCGGTATCGCTTACCTTG/GCAGACCCGCAATCATAAAC	
qFadD.F/R	TCTCCAGTCTGCATCTTTCC/CCATAGCCTTCCAGCAGATAC	
qFadE.F/R	TTACCCGTCTGGATGAACTG/GACGGCTTTCTTCAGCTTTC	
qFadL.F/R	GGGCGCTTCTATTACCTCTAA/TTTCAAGGTCGGTTGTACCC	
qRpoD.F/R	GAGCAAGGCTATCTGACCTATG/GCCCATGTCGTTGATCATTTG	

^a^Underlined sequences are homologous to the gene to be deleted

^b^Forward and reverse primers are denoted with an F or R, respectively, and primers used for qPCR are denoted with a q

### Gene deletions

The deletion of the *arcA* and *ompR* genes was accomplished using the λ red recombinase protocol, a commonly used method for nonpolar gene deletion, as previously described (Datsenko and Wanner 2000; Tappel et al. 2012b). Briefly, knockout cassettes were generated using PCR with gene-specific primers and the kanamycin resistance marker from pKD13 (Table 2). PCR was performed using PrimeSTAR HS polymerase (Takara) following the manufacturers recommended protocol. The λ red recombinase was expressed using plasmid pKD46, and knockout cassettes introduced by electroporation (1500 V, 5 ms; BTX ECM 399). Successfully recombination was determined by antibiotic selection and loci screening using check primers (Table 2). Antibiotic resistance was removed by the expression of FLP recombinase from the pCP20 plasmid, and successful deletions were confirmed by loss of antibiotic resistance and by PCR using loci check primers (Table 2). Deletion mutants *ΔarcA, ΔompR,* and the double deletion *ΔarcA ΔompR*) were named RSC02, RSC04, and RSC06, respectively (Table 2).

### PHA production

Protocols for PHA production and cell harvest were adapted from a previous study (Tappel et al. 2012b), with several modifications. For initial preliminary experiments, LSBJ, RSC02, RSC04, and RSC06 were made chemically competent and transformed with pBBR-C1J4SII following standard procedures (Sambrook and Russell 2001), to express PHA synthase and enoyl-CoA hydratase. Transformants were grown on LB-agar plates, and single colonies were used to inoculate separate 2 mL LB seed cultures, in triplicate for each strain. Seed cultures were grown for 16 h at 37 °C and 200 rpm, and used to inoculate 100 mL of growth media in 500 mL baffled shake flasks (to final concentration of 0.5%). Growth media contained LB, Brij-35, decanoic acid, and kanamycin. Cultures were grown for 48 h at 30 °C and 250 rpm on a rotary shaker, and were then harvested following previously published methods (Tappel et al. 2012b).

A more robust test of PHA homopolymer production was performed between LSBJ and RSC02 using the methods described above, with two key differences. A variety of fatty acids were tested in the growth media: sodium butyrate, pentanoic, hexanoic, heptanoic, sodium octanoate, decanoic, and dodecanoic. In addition, sodium phosphate dodecahydrate was added to the growth media for these experiments, which acts as a buffer for the shorter chain fatty acids. This addition also provides a significant source of phosphate to the media, so to keep growth conditions consistent, sodium phosphate dodecahydrate was added to every other experiment in this study.

### GC analysis

The yields and repeating unit compositions of PHA polymers were determined using GC, as previously described (Braunegg et al. 1978; Tappel et al. 2012b). Briefly, dried cells (15–20 mg) were dissolved in 2 mL of sulfuric acid: methanol solution (15:85) and 2 mL of chloroform and heated at 100 °C for 140 min in a 10 mL pressure vial (Kimax). The samples were cooled to room temperature followed by the addition of 1 mL of Nanopure filtered water, after which all samples were mixed by vortex. Aqueous and organic layers were allowed to separate for 20 min. The organic layer was passed through a 0.45 μm polytetrafluoroethylene (PTFE) syringe filter (Restek). An aliquot of 500 μL of each filtered sample was mixed with 500 μL of methyl octanoate standard (1 gL^−1^) in chloroform in a 2 mL GC vial. Samples were injected and separated using a GC 2010 Gas Chromatograph with an AOC-20i autoinjector with a flame ionization detector. Shimadzu’s GCSolution software was used to analyze the data, and statistical significance of triplicate samples was determined using a two-tailed Student’s *t* test with a 95% confidence interval (α = 0.05).

### Polymer purification and molecular weight determination

PHA homopolymers were extracted from residual dried cell samples from the LSBJ and RSC02 biosynthesis experiments by combining each set of triplicate samples into single 10 mL pressure vials (Kimax), adding 6 mL of chloroform, and incubating at 100 °C for 1 h. Each sample was filtered through a 0.45 μm PTFE syringe filter (Restek) into a 20 mL scintillation vial and rinsed twice with 2 mL aliquots of chloroform. Samples were concentrated to relative dryness using a rotary evaporator, and redissolved in 1 mL chloroform. Crude polymers were purified by non-solvent precipitation in cold methanol as described previously (Pinto et al. 2016), with several modifications. Briefly, dissolved samples were added dropwise to 10 mL of ice-cold methanol (4 °C) with rapid stirring. The solution was centrifuged (3452×*g*, 30 min, 4 °C) to pellet the PHA, decanted and washed with an additional 5 mL of methanol, and re-centrifuged. The supernatant was decanted, and the pellet dissolved in approximately 2 mL of chloroform to transfer to a scintillation vial. Samples were concentrated in a rotary evaporator, and evaporated to dryness under high vacuum for 4 h.

The weight average (M_w_) and number average (M_n_) molecular weights for each sample were determined by gel permeation chromatography (GPC) as described previously (Pinto et al. 2016). Briefly, PHA solutions of approximately 1.0 g L^−1^ were prepared by dissolution in chloroform and passed through a syringe filter (0.45 μm PTFE). Samples were injected (50 μL) into a Shimadzu LC-20AD liquid chromatograph equipped with a Shimadzu SIL-20A autosampler, a Shimadzu CTO-20A column oven, and a Shimadzu RID-10A refractive index detector. Samples were passed through an 8 × 50 mm styrenedivinylbenzene (SDV) guard column (5 μm particles; Polymer Standards Service) and an 8 × 300 mm SDV analytical column (5 μm particles; mixed bed porosity; max molecular weight 1E6 Da; Polymer Standards Service product sda0830051lim). The column oven was maintained at 40 °C with a 1 mL min^−1^ mobile phase of chloroform. Molecular weight standards of polystyrene with a narrow polydispersity index were used for calibration. Shimadzu’s LCsolution software was used to analyze the data. GPC chromatograms are available as supplemental material (Additional file 1: Figure S1).

### Growth profiles of LSBJ and RSC02

An analysis of the growth profiles for LSBJ and RSC02 was performed under the PHA homopolymer biosynthesis conditions utilizing two separate substrates, sodium butyrate and decanoic acid. Media and growth conditions were identical to those in the *PHA production* section. A 1 mL aliquot was removed from each culture to measure the OD_600_ every hour using a spectrophotometer (Genesys 10S) until the stationary phase was observed. A 5 mL aliquot was removed from each culture at both 24 and 48 h to analyze PHA concentrations. The 5 mL samples were harvested and analyzed by GC as described above.

### RNA extraction and qPCR analysis

RNA from *E. coli* LSBJ and RSC02 was isolated and purified as described previously (Lundgren et al. 2013; Sarwar et al. 2016). To isolate RNA for real-time quantitative PCR (qPCR), each strain was grown in duplicate in 100 ml of growth media (as described above, with sodium phosphate dodecahydrate and decanoic acid) in 500 ml baffled shake flasks at 30 °C and 250 rpm to an OD_600_ of ~0.6. Cultures were immediately stabilized by adding 1 ml of RNAprotect Bacteria reagent (Qiagen) to 0.5 ml of culture. Cells were then lysed with lysozyme and proteinase K as described in the manufacturer’s protocol. The total RNA was subsequently purified from the lysed cells with the RNeasy minikit (Qiagen) by using an on-column DNase digestion step. PCR and a Bioanalyzer were used to check the RNA for DNA contamination, quality, and concentration.

The iScript cDNA synthesis kit (Bio-Rad) was used to generate cDNA from 1 μg of the purified RNA samples. A tenfold dilution series of the pooled cDNA from the two duplicate RNA samples from LSBJ or RSC02 was used for the qPCR experiments. The qPCR experiments were performed in triplicate. The expression of several important genes for β-oxidation was normalized to *rpoD*, a housekeeping gene with stable expression during exponential growth (Jishage et al. 1996). Primers for qPCR were designed to produce ~100 bp amplicons of each of the following genes: *atoA, atoD, atoE, fadD, fadE, fadL,* and *rpoD* (Table 2). The qPCR mixtures contained 300 mM of each primer, 10 μl of the 2 × iQ SYBR green Supermix (Bio-Rad), 5 μl of diluted cDNA, and nuclease-free water to a total volume of 20 μl. qPCR was performed on the MiniOpticon system (Bio-Rad) with the following conditions: 1 cycle of 95 °C for 2 min and 40 cycles of 95 °C for 15 s, 55 °C for 30 s, and 72 °C for 30 s. Window-of-linearity *R* ^2^ values and amplification efficiency values ranged from 0.990 to 1.0 and 90.0% to >100%, respectively. The dilution series with the highest *R* ^2^ values was used to calculate relative gene expression of RSC02 compared to LSBJ using the Pfaffl method (Fleige et al. 2006). Amplification efficiencies and threshold cycle (Cq) values were calculated using the program LinRegPCR (Ruijter et al. 2009).

## Results

### PHA homopolymer production

**Fig. 2:**
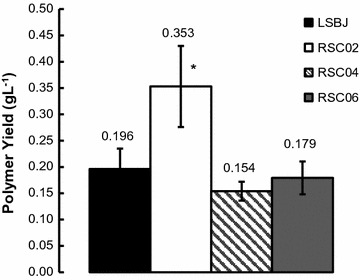
Comparison of poly(3-hydroxydecanoate) (PHD) yield between LSBJ and mutant strains RSC02, RSC04, and RSC06. The average yield achieved by RSC02 is significantly greater than that of LSBJ, while RSC04 and RSC06 are not significantly different from LSBJ. All values are averages of triplicate experiments plus or minus the standard deviation around those averages. An *asterisk* denotes a statistically significant difference compared to LSBJ (Student’s *t*-test, two-tailed, α = 0.05)

Our goal was to develop a strain capable of producing PHA polymers with controlled repeating unit compositions and increased yields. To achieve this, the mutant strains RSC02, RSC04, and RSC06 were derived from *E. coli* LSBJ by the deletion of *arcA*, *ompR,* and a tandem *arcA/ompR* deletion, respectively. The amount of poly(3-hydroxydecanoate) (PHD) produced by these strains was then compared to *E. coli* LSBJ while expressing PhaJ4 and PhaC1(STQK) in a set of preliminary experiments. RSC02 produced significantly more PHD than other strains, with a yield of 0.353 g L^−1^ (Fig. 2). Strains RSC04 and RSC06 were not found to be significantly different from LSBJ (Fig. 2; Additional file 1: Table S1).

To further investigate the effect of the Δ*arcA* mutation on PHA polymer production, PHA homopolymer biosynthesis was characterized utilizing a variety of fatty acids. The fatty acids used for the production of PHA homopolymers were sodium butyrate, valeric acid, hexanoic acid, heptanoic acid, sodium octanoate, decanoic acid, and dodecanoic acid respectively. Analysis by GC showed significant increases in the amount of polymer produced by *E. coli* RSC02 when compared to *E. coli* LSBJ for nearly all fatty acid substrates, particularly for those with six or fewer carbons (Table 3). Overall the parental strain, LSBJ, produced very little short chain-length PHAs, with yields of only 3.04 mg L^−1^ of poly(3-hydroxybutyrate) (PHB) and 1.92 mg L^−1^ of poly(3-hydroxyvalerate) (PHV), and only 44.8 mg L^−1^ of poly(3-hydroxyhexanoate) (PHHx), the shortest medium chain-length polymer (Table 3). We observed significant increases in the amount of PHA synthesized by RSC02 of approximately 3750, 6360, and 485% when cells utilized sodium butyrate, pentanoic acid, or hexanoic acid as substrates, respectively (Table 3). For these shorter chain-length PHA polymers the identity was confirmed by ^1^H-NMR due to the large differences in production between strains (Additional file 1: Figure S2). Of those PHA polymers tested with greater than 6 carbons per repeating unit, only poly(3-hydroxyheptanoate) (PHHp) and PHD yields were significantly different between strains, with an increase of approximately 61 and 115 percent observed for RSC02, respectively (Table 3).

**Table 3:** PHA yield comparison between LSBJ and RSC02

Substrate	Strain	CDW (gL^−1^)^a^	PHA^a^ (wt%)	PHA concentration (mg L^−1^)^a^	Percent increase^b^
Sodium butyrate	LSBJ	0.80 ± 0.03	0.38 ± 0.05	3.04 ± 0.27	3750
	RSC02	0.85 ± 0.03	13.7 ± 0.95	117 ± 11.1^*^	
Pentanoic acid	LSBJ	0.76 ± 0.05	0.23 ± 0.02	1.92 ± 0.42	6360
	RSC02	1.14 ± 0.04^*^	11.1 ± 1.01	124 ± 7.25^*^	
Hexanoic acid	LSBJ	0.85 ± 0.01	5.47 ± 0.97	44.8 ± 6.13	485
	RSC02	1.06 ± 0.01^*^	27.3 ± 4.28	262 ± 59.0^*^	
Heptanoic acid	LSBJ	0.93 ± 0.01	23.4 ± 0.48	198 ± 4.27	61
	RSC02	1.02 ± 0.06	30.2 ± 2.28	319 ± 22.8^*^	
Sodium octanoate	LSBJ	1.22 ± 0.05	44.5 ± 8.68	543 ± 110	1.10
	RSC02	1.03 ± 0.01^*^	54.1 ± 1.06	549 ± 12.5	
Decanoic acid	LSBJ	1.23 ± 0.05	29.3 ± 2.15	281 ± 152	115
	RSC02	1.49 ± 0.02^*^	40.4 ± 1.30	603 ± 26.4^*^	
Dodecanoic acid	LSBJ	1.31 ± 0.17	23.5 ± 5.42	303 ± 38.4	7.26
	RSC02	1.11 ± 0.03^*^	29.3 ± 5.21	325 ± 64.0	

^*^Denotes statistically significant difference compared to LSBJ (Student’s *t*-test, two-tailed, α = 0.05)

^a^All values are averages of biological triplicate experiments plus or minus the standard deviation about those averages

^b^Percent increase calculated as the increase in PHA concentration from RSC02 compared to LSBJ

### Molecular weight comparison

To compare differences in physical properties of the polymers synthesized by *E. coli* LSBJ and RSC02, samples were analyzed by gel permeation chromatography (GPC) to determine the number average molecular weight (M_n_), the weight average molecular weight (M_w_), and the polydispersity (M_w_/M_n_) (Table 4). The polymers extracted from RSC02 had greater molecular weights than those from LSBJ for every polymer except poly(3-hydroxydodecanoate) (PHDD), which had a M_n_ of approximately 60 kDa and a M_w_ of approximately 172 kDa for both strains (Table 4). In addition, the polymers extracted from RSC02 had a greater degree of polydispersity, again with the exception of PHDD (Table 4).

**Table 4:** PHA molecular weight data

PHA^a^	Strain	Mw (kDa)	Mn (kDa)	Mw/Mn
PHB	LSBJ	ND	ND	ND
	RSC02	390	117	3.3
PHV	LSBJ	18	16	1.2
	RSC02	243	79	3.1
PHHx	LSBJ	134	77	1.7
	RSC02	408	171	2.4
PHHp	LSBJ	219	106	2.1
	RSC02	319	134	2.4
PHO	LSBJ	157	73	2.1
	RSC02	285	99	2.9
PHD	LSBJ	145	50	2.9
	RSC02	234	67	3.5
PHDD	LSBJ	173	58	3.0
	RSC02	172	60	2.9

^a^ *PHB* poly(3-hydroxybutyrate); *PHV* poly(3-hydroxyvalerate); *PHHx* poly(3-hydroxyhexanoate); *PHHp* poly(3-hydroxyheptanoate); *PHO* poly(3-hydroxyoctanoate); *PHD* poly(3-hydroxydecanoate); *PHDD* poly(3-hydroxydodecanoate); *ND* not detected

### Growth profile of LSBJ and RSC02

**Fig. 3:**
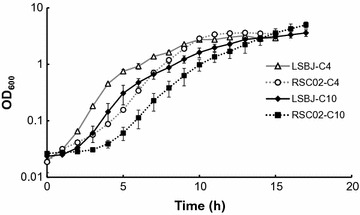
Growth profile of *E. coli* LSBJ and RSC02 during PHA-biosynthesis utilizing sodium butyrate (C4) or decanoic acid (C10). For both substrates, RSC02 displayed an extended lag time and slightly slower growth rate relative to LSBJ; however, RSC02 cultures reached approximately the same density as LSBJ upon reaching the stationary phase. For both strains, growth with sodium butyrate as the substrate caused a shorter lag phase than with decanoic acid substrate. All values are the averages of triplicate experiments plus or minus the standard deviation around those averages

We visually observed that RSC02 cultures appeared less optically dense than those of LSBJ during the first day of growth, which would be indicative of an increased lag time for this strain. To quantify this observation, we investigated the growth profiles of LSBJ and RSC02 under PHA biosynthesis conditions supplemented with either sodium butyrate or decanoic acid. There was both an increase in lag time and a decrease in the growth rate of RSC02 relative to LSBJ for both substrates (Fig. 3). Regardless of strain, when the fatty acid substrate was sodium butyrate the lag phase was considerably shorter and the stationary phase was reached more rapidly (Fig. 3). Despite the increased lag phase duration and slightly slower growth rate, RSC02 reached the same culture density as LSBJ when supplemented with sodium butyrate (OD_600_ ~ 3.5, 15 h), and reached a higher OD_600_ when supplemented with decanoic acid of ~5.0 compared to ~3.6 in LSBJ by hour 17 (Fig. 3).

### PHA yield at 24 vs 48 h

**Fig. 4:**
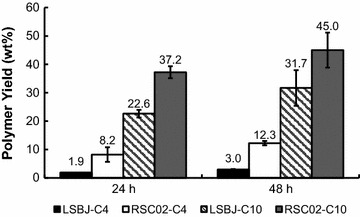
Comparison of PHA yield as a percentage of dry weight between LSBJ and RSC02 at 24 h and 48 h, utilizing either sodium butyrate (C4) or decanoic acid (C10). LSBJ saw insignificant changes in PHB production between 24 and 48 h, while RSC02 increased slightly from 8.2 to 12.3% of cell dry weight. PHD yield from LSBJ increased from 22.6 to 31.7% between 24 and 48 h, an increase of ~40%, while PHD yield from RSC02 increased from 37.2 to 45.0%, an increase of ~20%. Regardless of the time, RSC02 produced more polymer than LSBJ in all cases. All values are the averages of triplicate experiments plus or minus the standard deviation about those averages

To determine whether the increased lag phase in RSC02 had a negative effect on PHA yield earlier in the production cycle, small subsamples were removed from both LSBJ and RSC02 shake flasks after 24 h and 48 h during the growth profile experiments. Similar to the data shown in Table 3, LSBJ produced very little PHB at either 24 or 48 h, and there was very little change between the two time points (Fig. 4). However, LSBJ yields of PHD increased from 22.6 to 31.7% of cell dry weight, an increase of approximately 40% (Fig. 4). RSC02 produced significantly more polymer than LSBJ regardless of the time or fatty acid substrate; PHB yield increased from 8.2 to 12.3% from 24 to 48 h, while PHD yield increased by ~20% between time points from 37.2 to 45.0% (Fig. 4).

### Relative gene expression of RSC02

**Fig. 5:**
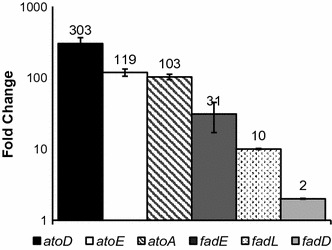
Relative gene expression of RSC02 compared to LSBJ, normalized to *rpoD* and measured as fold changes. The expression of genes related to fatty acid degradation was significantly increased in the RSC02 strain during mid-exponential growth phase (OD_600_ of ~0.6). The SCL fatty acid degradation genes *atoA, atoE,* and *atoD* were massively upregulated by 103-fold, 119-fold, and 303-fold respectively. MCL fatty acid degradation genes *fadD, fadL,* and *fadE* were upregulated by twofold, tenfold, and 31-fold respectively. Relative gene expression was calculated from qPCR fluorescence data using the LineRegPCR software (Ruijter et al. 2009) to calculate amplification efficiency and Cq values, and the Pfaffl method used to derive relative expression values with LSBJ as the calibrator (Fleige et al. 2006). All values are the averages of triplicate experiments plus or minus the standard deviation about those averages

To analyze the relative expression of fatty acid degradation genes, RNA was isolated from mid-exponential phase cultures of LSBJ and RSC02 and reverse transcribed into cDNA for qPCR. An appropriate OD_600_ value (~0.6) for mid-exponential phase was determined using the previously defined growth profile (Fig. 3). All the genes analyzed were upregulated to some degree in RSC02; SCL fatty acid uptake genes *atoA, atoE* and *atoD* were all upregulated to an extremely large degree (>100-fold), while *fadD, fadE,* and *fadL* were all upregulated to a lesser degree (2-, 10-, and 31-fold) (Fig. 5). It is important to note that the expression values reported for the *ato* system may be exaggerated due to the low number of transcripts observed for LSBJ (as observed by a late-cycle emergence of the fluorescence during qPCR analysis).

## Discussion

### PHA homopolymer production

The purpose of developing the mutant strains RSC02, RSC04, and RSC06 was to improve PHA biosynthesis by removing regulatory genes known to interact with components of the PHA biosynthesis pathway. Based on the evidence seen in Fig. 2, the singular deletion of *arcA* conferred a significant increase in the production of PHD compared to the parental LSBJ. A reasonable explanation for this observation is that ArcA becomes active in *E. coli* LSBJ during the growth conditions utilized for polymer production and inhibits the transcription of *fadL*, *fadD*, and *fadE*, which are known targets of ArcA regulation (Table 1). In an *arcA* deletion mutant this inhibition cannot occur, likely resulting in higher basal transcription of these *fad* genes. The deletion of *ompR* and the double deletion of *ompR/arcA* provided no benefit to PHD biosynthesis, and no statistically significant differences were observed between these mutants and LSBJ (Fig. 2). One possibility for this apparent lack of effect is that OmpR regulatory target FadL is not active towards decanoic acid, which has been demonstrated previously (Black 1990). However, the similarity between the RSC06 double deletion mutant and the RSC04 mutant suggests another possibility; the loss of regulation by OmpR may yield a mildly toxic phenotype that inhibits cell growth and metabolism. This possibility is supported by both the observation that both RSC04 and RSC06 had significantly lower cell dry weights than either LSBJ and RSC02 (Additional file 1: Table S1), and by previous reporting from Oshima et al. (2002) that showed a marked growth deficiency in *E. coli* Δ*ompR* mutants. These findings provided motivation for further characterization of the RSC02 mutant.

A variety of fatty acid substrates were used to characterize the differences in PHA homopolymer production between RSC02 and LSBJ. In nearly all cases, the deletion of *arcA* resulted in a significantly higher polymer yield (Table 3). Most surprisingly, the largest increases in polymer yield observed between RSC02 and LSBJ were for the two SCL polymers, PHB and PHV, as well as the shortest MCL polymer, PHHx (Table 3). This result is interesting considering that there are fewer steps involved in SCL fatty acid metabolism that are regulated by ArcA. Short chain fatty acids enter the β-oxidation cycle via enzymes derived from the *ato* operon, bypassing both FadL and FadD (Fig. 1). This operon has no known interaction with ArcA and is instead regulated by the response regulator AtoC, which is present in both LSBJ and RSC02 as a constitutively expressed mutant that confers a high level of *atoDAEB* transcription (Spratt et al. 1981; Jenkins and Nunn 1987; Kyriakidis and Tiligada 2009). One possible explanation for this drastic difference in SCL PHA production is that secondary metabolite pools are different between the two strains; polyamines for example have been shown to have significant effects on the regulation of AtoC (Kyriakidis and Tiligada 2009), and previous studies have shown that aspects of polyamine metabolism are affected by the deletion of *arcA* (Partridge et al. 2006).

The differences in MCL PHA production between LSBJ and RSC02 are more varied than those observed for SCL polymers. No significant difference was found between strains when comparing the yields of poly(3-hydroxyoctanoate) (PHO) and PHDD; however, the yields of poly(3-hydroxyheptanoate) (PHHp) and PHD were significantly increased in RSC02 (Table 3). The most reasonable explanation for this is that there are numerous differences in the substrate specificities of the enzymes involved in the PHA biosynthesis pathway. The long chain fatty acid transporter FadL is predominantly active on fatty acids containing 16 or more carbons, and no binding has been observed for decanoic acid, therefore it is unlikely that changes in *fadL* expression would account for the differences we observed (Black 1990). In contrast, the acyl-CoA synthetase FadD has high activity towards 12 and 10 carbon fatty acids and only low activity for 8 and 6 carbon fatty acids (Iram and Cronan 2006; Ford and Way 2015), while the recombinant enzymes PhaJ4 and PhaC1(STQK) each have their own well-documented substrate specificities that could contribute to the observed variation without any direct regulation by ArcA (Tsuge et al. 2003; Matsumoto et al. 2005; Sato et al. 2011). However, a complete understanding of fatty acid flux through this PHA biosynthesis pathway cannot be achieved with the current lack of information regarding the enzymatic activity of the acyl-CoA dehydrogenase FadE.

### Comparison of the molecular weights of PHAs produced by LSBJ and RSC02

The molecular weight data showed a great degree of variability between PHAs, as well as between LSBJ and RSC02, with polydispersity values (M_w_/M_n_) from 1.2 to 3.3 (Table 4). The molecular weight of polymers produced by RSC02 were higher for all PHA polymers produced except for PHDD, and in general the polydispersity indices were higher for RSC02-derived polymers as well. Both the PHO and PHD molecular-number-average-molecular weights (M_n_) were substantially lower for LSBJ than in previous studies, while the molecular weight and polydispersity for PHDD was found to be significantly higher than previously reported (Liu et al. 2011; Tappel et al. 2012b). A reliable comparison of the molecular weights observed in this study to other studies is difficult due to the variety of growth conditions and pathways employed. However, a possible explanation for the differences observed between LSBJ and RSC02 is that increased basal expression of β-oxidation genes improves the supply of 3-hydroxy fatty acyl-CoA monomers to the PhaC1 (STQK) polymer synthase. The evidence summarized in Table 4 suggests that the molecular machinery in *E. coli* plays some role in determining the molecular weight of the polymers produced.

A comparison between the polymer yield data in Table 3 and the molecular weight data in Table 4 appears to support this explanation. The PHA homopolymers with the greatest increases in yield (PHB, PHV, and PHHx) similarly showed the greatest increases in molecular weight (with the partial exception of PHB, which was not extracted in a great enough quantity to detect by GPC). The PHHp and PHD obtained from RSC02, which also saw moderate improvement in terms of yield, was observed to have a higher molecular weight than that obtained from LSBJ, although to a lesser extent than the shorter chain length PHA homopolymers. Interestingly, PHO produced by RSC02 had a higher molecular weight despite there being no significant difference in yield, while PHDD was not significantly different in either of those measurements. While it appears that improving the utilization of fatty acids also improves PHA molecular weight using this biosynthetic platform, these minor discrepancies with regards to PHO reveal that this relationship is more complex than that.

Another possible explanation for this increase in molecular weights could be that less ethanol is produced by RSC02 than LSBJ. It is typical for bacterial cultures grown into stationary phase in shake flasks to reach some level of microaerobiosis, leading to the production of fermentative byproducts such as ethanol (Gupta and Rao 2003; Losen et al. 2004). It was previously reported that the supplementation of ethanol in cultures of recombinant *E. coli* led to a decrease in the molecular weight of PHB due to a chain transfer reaction from PhaC to ethanol (Hiroe et al. 2013). In *E. coli*, the reversible enzyme AdhE is largely responsible for ethanol flux within the cell, allowing for both the biosynthesis and degradation of ethanol (Membrillo-Hernandez et al. 2000). It has been previously shown that an Δ*arcA* mutant has an improved ethanol tolerance compared to wild type, and it has been hypothesized that this tolerance is derived from increased expression of *adhE* along with enzymes involved in the tricarboxylic acid cycle (TCA), which could drive the breakdown of ethanol to acetyl-CoA (Goodarzi et al. 2010). There is also evidence in the literature showing that *adhE* expression is significantly higher in an *E. coli* Δ*arcA* mutant than in wild type under a range of microaerobic conditions (Shalel-Levanon et al. 2005). It is therefore possible that endogenously produced ethanol is reducing PHA molecular weights via chain termination in LSBJ, and that this effect could be mitigated by an improved flux of ethanol back to acetyl-CoA. However, further investigation of this possibility is required as there is currently no direct evidence to support this explanation.

### Growth profiles of LSBJ and RSC02

We investigated our observation that RSC02 had slower growth than LSBJ by recording hourly culture densities, and found that while RSC02 had a significantly longer lag phase, the growth rate was nearly identical to LSBJ, with cultures reaching similar final cell densities regardless of which fatty acid substrate was provided (Fig. 3). These observations match the evidence found in the Keio collection of single-gene knockouts, which showed that *arcA* is nonessential, an Δ*arcA* mutant grows only slightly slower than wild type, and reaches only marginally lower culture densities (Baba et al. 2006). Despite the slightly slower growth observed with RSC02, the strain is capable of producing significantly more polymer than LSBJ even after only 24 h of growth (Fig. 4). This offers an advantage when using this strain for a large-scale continuous fermentation, and shows that the lengthened lag phase does not significantly impede PHA biosynthesis.

### Relative gene expression of RSC02

Our analysis of the qPCR results shows a clear increase in the expression of each of the fatty acid degradation genes tested (Fig. 5). Although the improvements to *fadD, fadL,* and *fadE* were expected and support our hypothesis, the magnitude of the increase in the three *ato* genes came as a surprise. As we mentioned previously, ArcA is not known to directly regulate expression of the *ato* system, and both LSBJ and RSC02 harbor a constitutively expressed mutant *atoC* gene (Spratt et al. 1981; Jenkins and Nunn 1987; Kyriakidis and Tiligada 2009). Comparing the drastic differences seen in SCL PHA yields between LSBJ and RSC02 provides strong evidence to suggest that the similarly drastic increases in gene expression are responsible for these effects (Table 3). These results support the idea that the Δ*arcA* mutation indirectly effects the expression of the *ato* system, possibly mediated by an altered polyamine metabolism.

The differences between the relative expression of *fadD, fadL* and *fadE* also raise some interesting observations. For example, *fadD* was only modestly up-regulated (twofold) and its protein product has a high activity towards both 10 and 12 carbon fatty acid substrates; however, while PHD biosynthesis was significantly improved PHDD biosynthesis was not (Table 3; Fig. 5). Similarly, *fadL* expression was significantly increased in RSC02 (tenfold) which does have limited binding affinity for 12 carbon fatty acids (Black 1990), and yet this does not appear to improve PHDD biosynthesis either. These results suggest that the binding affinity of FadE may be an important limiting factor in PHA biosynthesis using this system, considering that *fadE* was the most highly up-regulated of these three genes (31-fold) and yet only the yields of PHHx, PHHp, and PHD were significantly improved (Table 3; Fig. 5). While the binding affinity of FadE is not well-known, our results suggest that it may have low binding affinity towards octanoyl-CoA and dodecanoyl-CoA, although further investigation is needed to test that hypothesis.

## Conclusion

One of the largest challenges still facing the PHA industry is the relatively low yield of polymer obtained, which contributes to the overall cost (Kaur 2015). The strain *E. coli* RSC02 that was developed in this study offers a significant improvement to the previously reported strain, *E. coli* LSBJ. The most significant improvements were seen in the biosynthesis of PHB, PHV, and PHHx, with modest increases observed in PHHp and PHD. These results are supported by the increased expression of *atoA, atoD,* and *atoE* which correlates with improved PHB and PHV biosynthesis, and also the increased expression of *fadD, fadL,* and *fadE* which may contribute to the increased biosynthesis of PHHx, PHHp, and PHD.

## Additional file

**Additional file 1: Figure S1A.** GPC chromatogram of purified poly(3-hydroxybutyrate) (C4) produced by RSC02, plotted as normalized intensity vs retention time. No data available for LSBJ. **Figure S1B, C** GPC chromatogram of purified poly(3-hydroxyvalerate) (C5) produced by LSBJ (B) and RSC02 (C), plotted as normalized intensity vs retention time. **Figure S1D, E** GPC chromatogram of purified poly(3-hydroxyhexanoate) (C6) produced by LSBJ (D) and RSC02 (E), plotted as normalized intensity vs retention time. **Figure S1F, G** GPC chromatogram of purified poly(3-hydroxyheptanoate) (C7) produced by LSBJ (F) and RSC02 (G), plotted as normalized intensity vs retention time. **Figure S1H, I** GPC chromatogram of purified poly(3-hydroxyoctanoate) (C8) produced by LSBJ (H) and RSC02 (I), plotted as normalized intensity vs retention time. **Figure S1J, K** GPC chromatogram of purified poly(3-hydroxydecanoate) (C10) produced by LSBJ (J) and RSC02 (K), plotted as normalized intensity vs retention time. **Figure S1L, M** GPC chromatogram of purified poly(3-hydroxydodecanoate) (C12) produced by LSBJ (L) and RSC02 (M), plotted as normalized intensity vs retention time. **Figure S2A** 1H-NMR (600 MHz, CDCl3); δ 5.28-5.23 (sext, 1H), 2.62-2.45 (m, 2H), 1.28-1.25 (d, 3H). **Figure S2B** 1H-NMR (600 MHz, CDCl3); δ 5.17-5.13 (p, 1H), 2.59-2.50 (m, 2H), 1.66-1.59 (m, 2H), 0.91-0.88 (t, 3H). The asterisk (*) at δ 1.53 denotes a water impurity (Fulmer et al. 2010). **Figure S2C** 1H-NMR (600 MHz, CDCl3); δ 5.21-5.18 (p, 1H), 2.59-2.48 (m, 2H), 1.60-1.54 (m, 2H), 1.35-1.27 (m, 2H), 0.91 (t, 3H). The asterisk (*) at δ 1.53 denotes a water impurity (Fulmer et al. 2010). **Table S1**: Poly(3-hydroxydecanoate) yield comparison between LSBJ, RSC02, RSC04.
