# Supplementary material for: Enhancing poly(3-hydroxyalkanoate) production in Escherichia coli by the removal of the regulatory gene arcA
Source: AMB Express. 2016 Nov 23;6:120. doi: 10.1186/s13568-016-0291-z (PMC5120623; doi:10.1186/s13568-016-0291-z)
Supplement: Supplementary file 1 — Additional file 1: Figure S1A. GPC chromatogram of purified poly(3-hydroxybutyrate) (C4) produced by RSC02, plotted as normalized intensity vs retention time. No data available for LSBJ. Figure S1B, C GPC chromatogram of purified poly(3-hydroxyvalerate) (C5) produced by LSBJ (B) and RSC02 (C), plotted as normalized intensity vs retention time. Figure S1D, E GPC chromatogram of purified poly(3-hydroxyhexanoate) (C6) produced by LSBJ (D) and RSC02 (E), plotted as normalized intensity vs retention time. Figure S1F, G GPC chromatogram of purified poly(3-hydroxyheptanoate) (C7) produced by LSBJ (F) and RSC02 (G), plotted as normalized intensity vs retention time. Figure S1H, I GPC chromatogram of purified poly(3-hydroxyoctanoate) (C8) produced by LSBJ (H) and RSC02 (I), plotted as normalized intensity vs retention time. Figure S1J, K GPC chromatogram of purified poly(3-hydroxydecanoate) (C10) produced by LSBJ (J) and RSC02 (K), plotted as normalized intensity vs retention time. Figure S1L, M GPC chromatogram of purified poly(3-hydroxydodecanoate) (C12) produced by LSBJ (L) and RSC02 (M), plotted as normalized intensity vs retention time. Figure S2A 1H-NMR (600 MHz, CDCl3); δ 5.28-5.23 (sext, 1H), 2.62-2.45 (m, 2H), 1.28-1.25 (d, 3H). Figure S2B 1H-NMR (600 MHz, CDCl3); δ 5.17-5.13 (p, 1H), 2.59-2.50 (m, 2H), 1.66-1.59 (m, 2H), 0.91-0.88 (t, 3H). The asterisk (*) at δ 1.53 denotes a water impurity (Fulmer et al. 2010). Figure S2C 1H-NMR (600 MHz, CDCl3); δ 5.21-5.18 (p, 1H), 2.59-2.48 (m, 2H), 1.60-1.54 (m, 2H), 1.35-1.27 (m, 2H), 0.91 (t, 3H). The asterisk (*) at δ 1.53 denotes a water impurity (Fulmer et al. 2010). Table S1: Poly(3-hydroxydecanoate) yield comparison between LSBJ, RSC02, RSC04. [file 13568_2016_291_MOESM1_ESM.pdf]

Enhancing poly(3-hydroxyalkanoate) production in *Escherichia coli* by the removal of the regulatory gene *arcA*

Journal of Applied Microbiology and Biotechnology

Ryan A. Scheel[1], Liyuan Ji[1], Benjamin R. Lundgren[1], Christopher T. Nomura[1,2,3,#]

[1]*Department of Chemistry and* [2]*Center for Applied Microbiology, State University of New York College of Environmental Science and Forestry, 1 Forestry Drive, 13210 Syracuse, NY, USA*

[3]*Hubei Collaborative Center for Green Transformation of Bio-Resources, College of Life Sciences, Hubei University, Wuhan 430062, China*

[#]*Corresponding Author: ctnomura@esf.edu, tel. 1-315-470-6854, fax 1-315-470-6856*

The weight average (Mw) and number average (Mn) molecular weights for each sample were determined by gel permeation chromatography (GPC) as described previously (Pinto et al. 2016). Briefly, PHA solutions of approximately 1.0 g L-1 were prepared by dissolution in chloroform and passed through a syringe filter (0.45 µm PTFE). Samples were injected (50 µL) into a Shimadzu LC-20AD liquid chromatograph equipped with a Shimadzu SIL-20A autosampler, a Shimadzu CTO-20A column oven, and a Shimadzu RID-10A refractive index detector. Samples were passed through an 8 x 50 mm styrenedivinylbenzene (SDV) guard column (5 µm particles; Polymer Standards Service) and an 8 x 300 mm SDV analytical column (5 µm particles; mixed bed porosity; max molecular weight 1E6 Da; Polymer Standards Service product sda0830051lim). The column oven was maintained at 40°C with a 1 mL min-1 mobile phase of chloroform. Molecular weight standards of polystyrene with a narrow polydispersity index were used for calibration. Shimadzu's LCsolution software was used to analyze the data, and chromatograms were generated using RStudio and compiled using Adobe Illustrator.

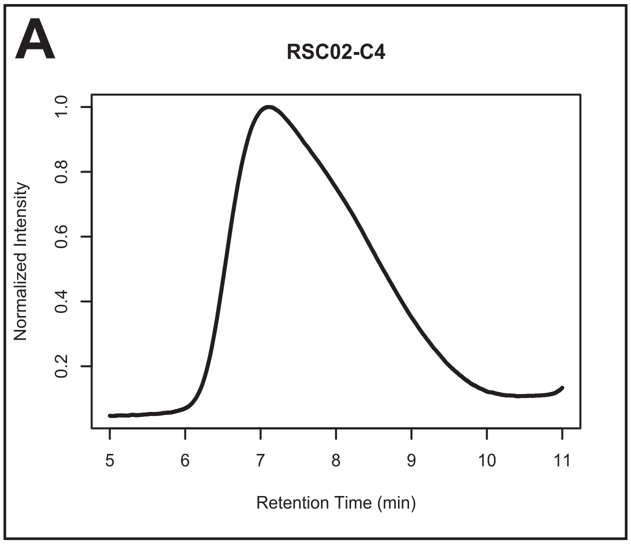

Figure S1-A GPC chromatogram of purified poly(3-hydroxybutyrate) (C4) produced by RSC02, plotted as normalized intensity vs retention time. No data available for LSBJ.

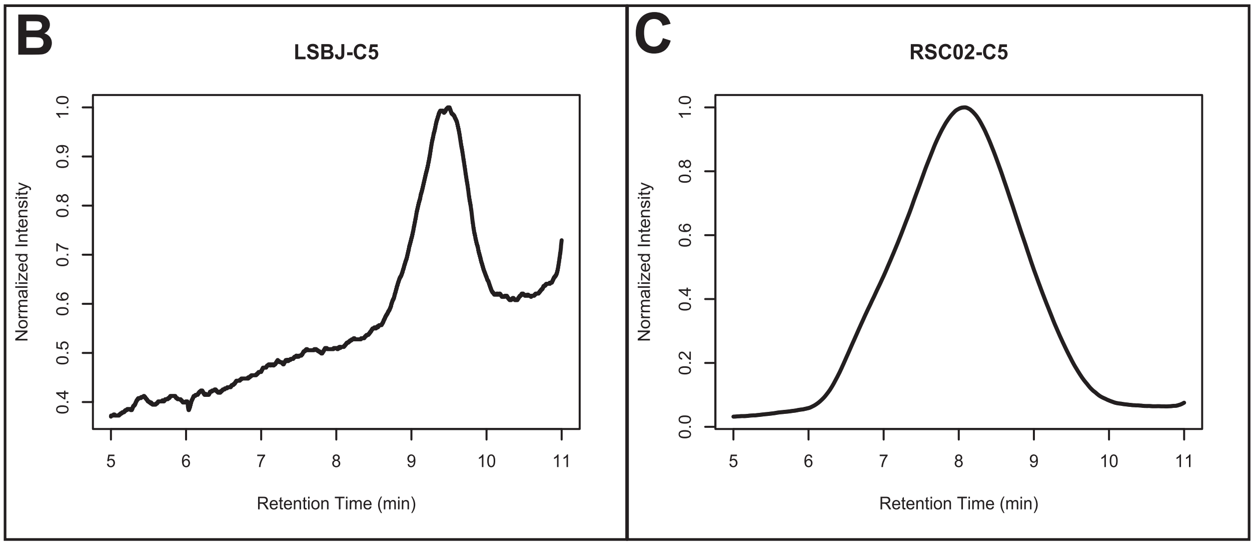

Figure S1-BC GPC chromatogram of purified poly(3-hydroxyvalerate) (C5) produced by LSBJ (B) and RSC02 (C), plotted as normalized intensity vs retention time.

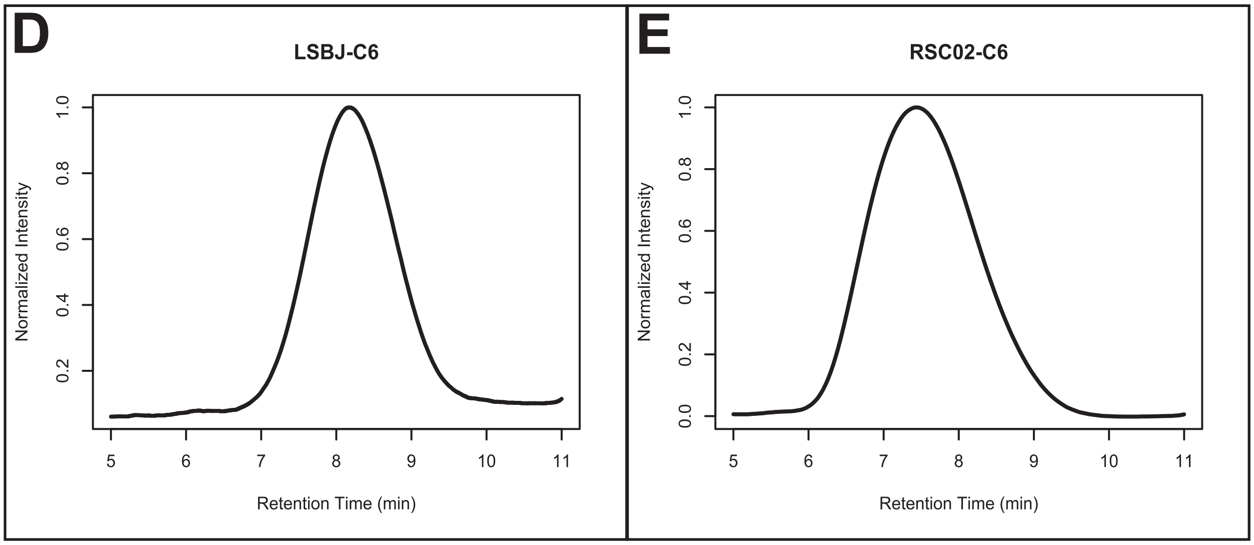

Figure S1-DE GPC chromatogram of purified poly(3-hydroxyhexanoate) (C6) produced by LSBJ (D) and RSC02 (E), plotted as normalized intensity vs retention time.

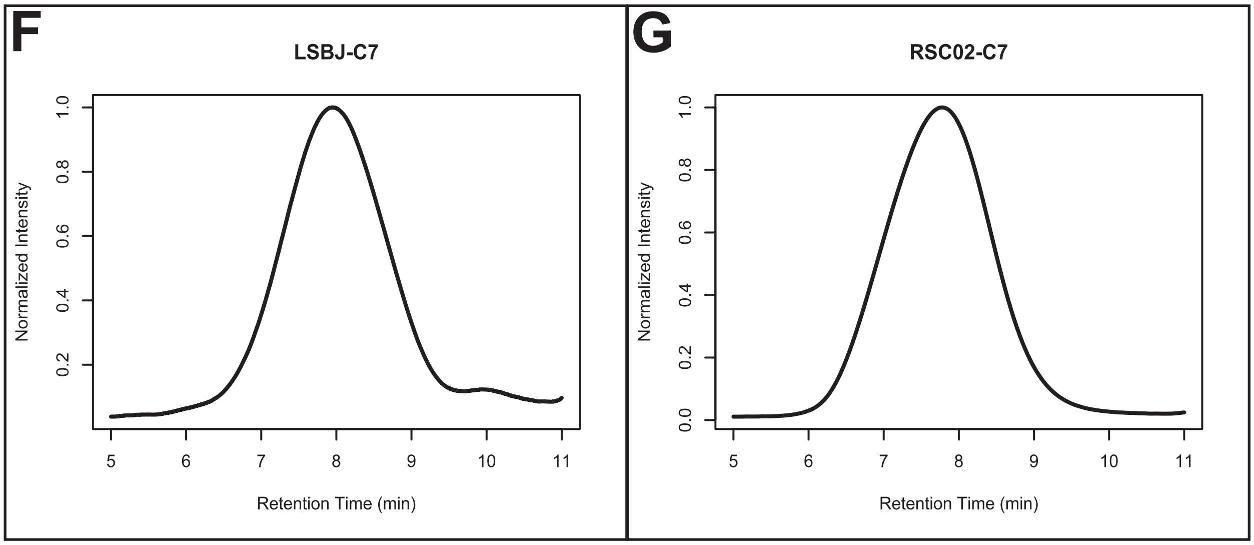

Figure S1-FG GPC chromatogram of purified poly(3-hydroxyheptanoate) (C7) produced by LSBJ (F) and RSC02 (G), plotted as normalized intensity vs retention time.

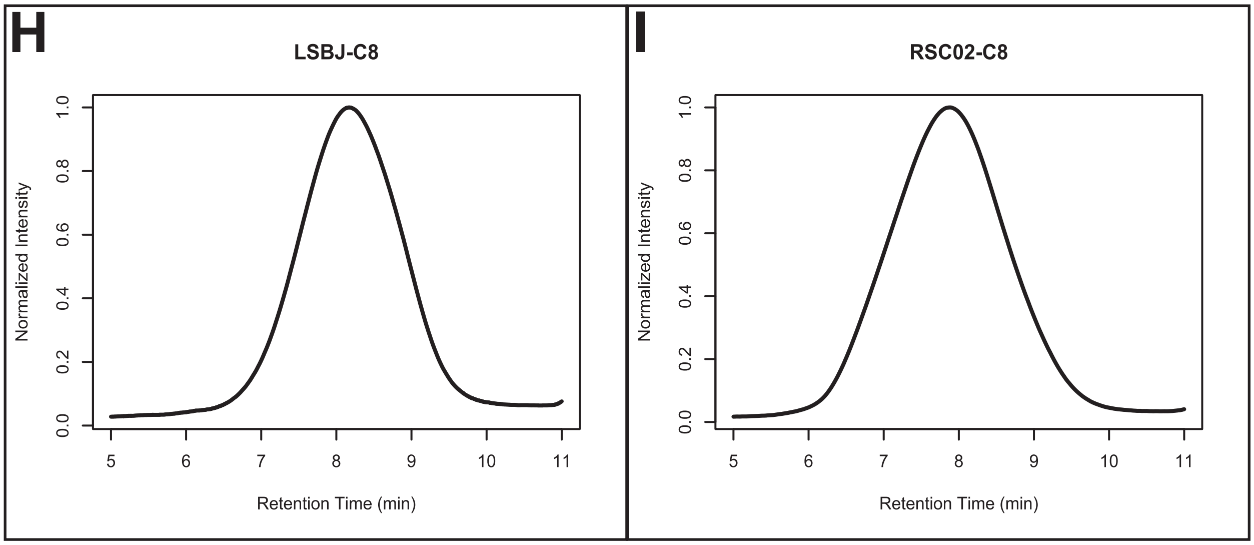

Figure S1-HI GPC chromatogram of purified poly(3-hydroxyoctanoate) (C8) produced by LSBJ (H) and RSC02 (I), plotted as normalized intensity vs retention time.

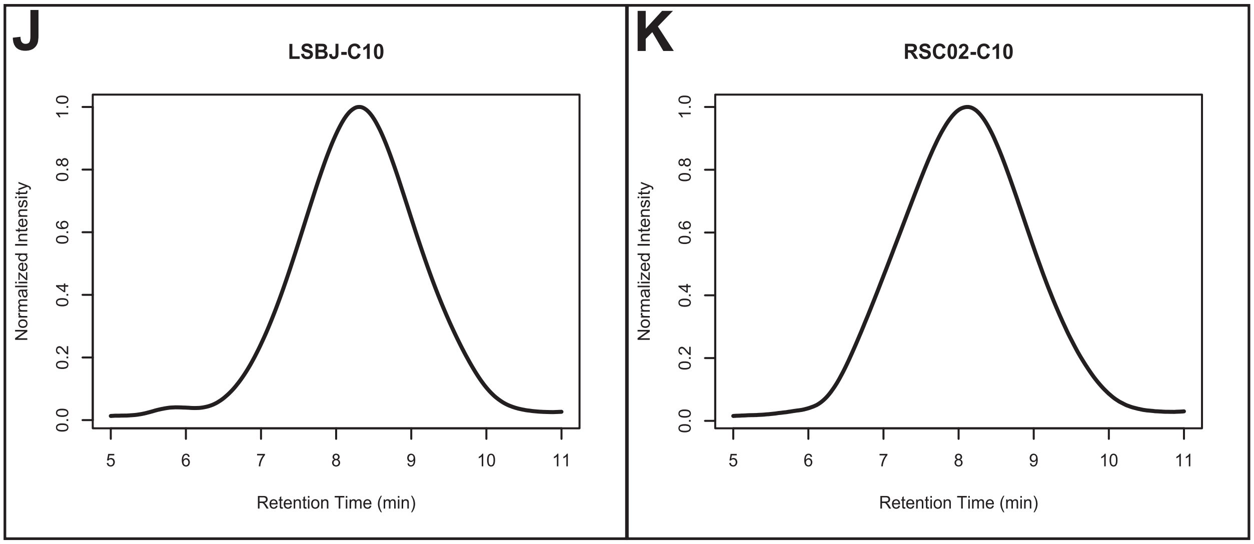

Figure S1-JK GPC chromatogram of purified poly(3-hydroxydecanoate) (C10) produced by LSBJ (J) and RSC02 (K), plotted as normalized intensity vs retention time.

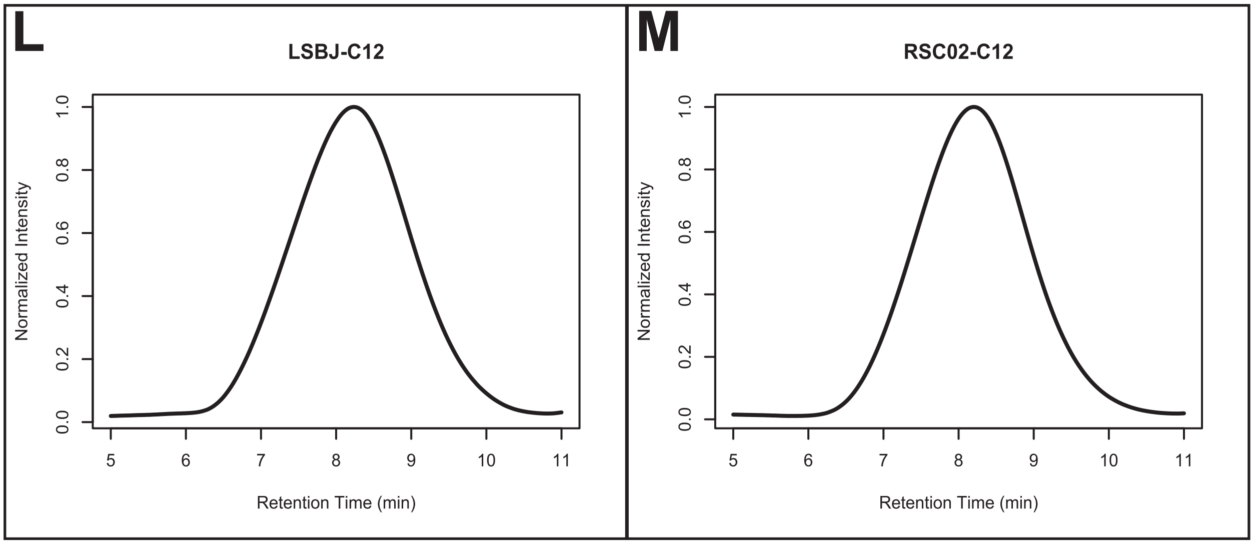

Figure S1-LM GPC chromatogram of purified poly(3-hydroxydodecanoate) (C12) produced by LSBJ (L) and RSC02 (M), plotted as normalized intensity vs retention time.

NMR spectra were recorded on a Bruker AVANCE III 600 MHz instrument, and were calibrated using residual undeuterated solvents as internal reference (chloroform, δ = 7.26 ppm, ^{1}H NMR). Chemical shifts (δ) are reported in parts per million (ppm); NMR peak multiplicities are denoted by the following abbreviations: s = singlet, d = doublet, t = triplet, q = quartet, p = pentet, sext = sextet, dd = doublet of doublets, dt = doublet of triplets, m = multiplet, br = broad. Spectra were processed with Bruker TopSpin v3.5pI2.

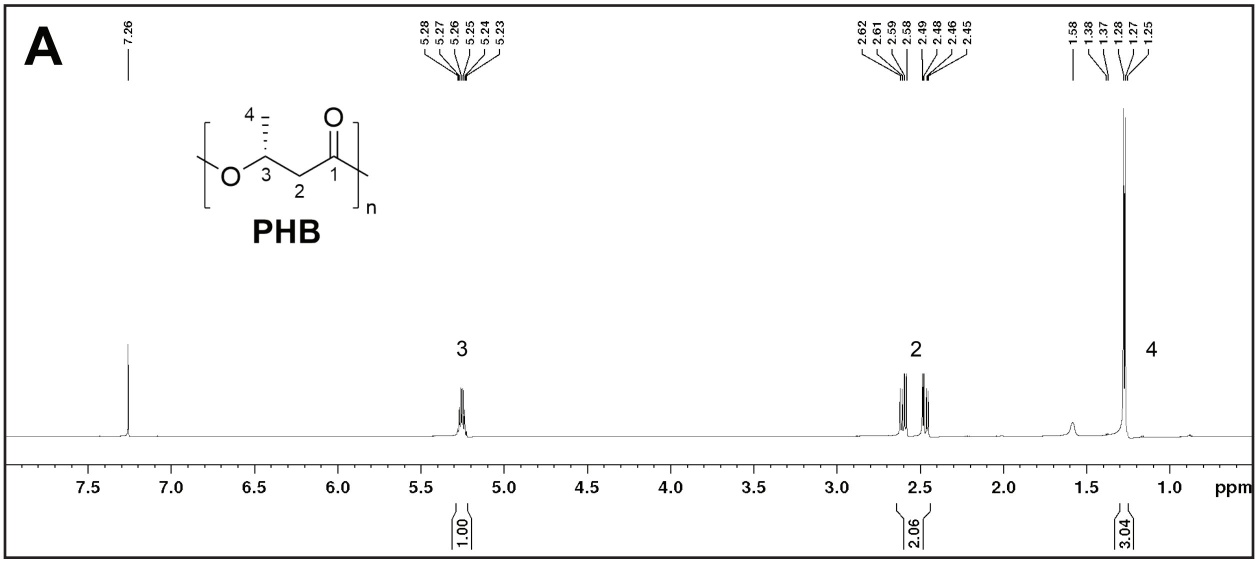

Figure S2-A ^{1}H-NMR (600 MHz, $CDCl_3$); δ 5.28-5.23 (sext, 1H), 2.62-2.45 (m, 2H), 1.28-1.25 (d, 3H).

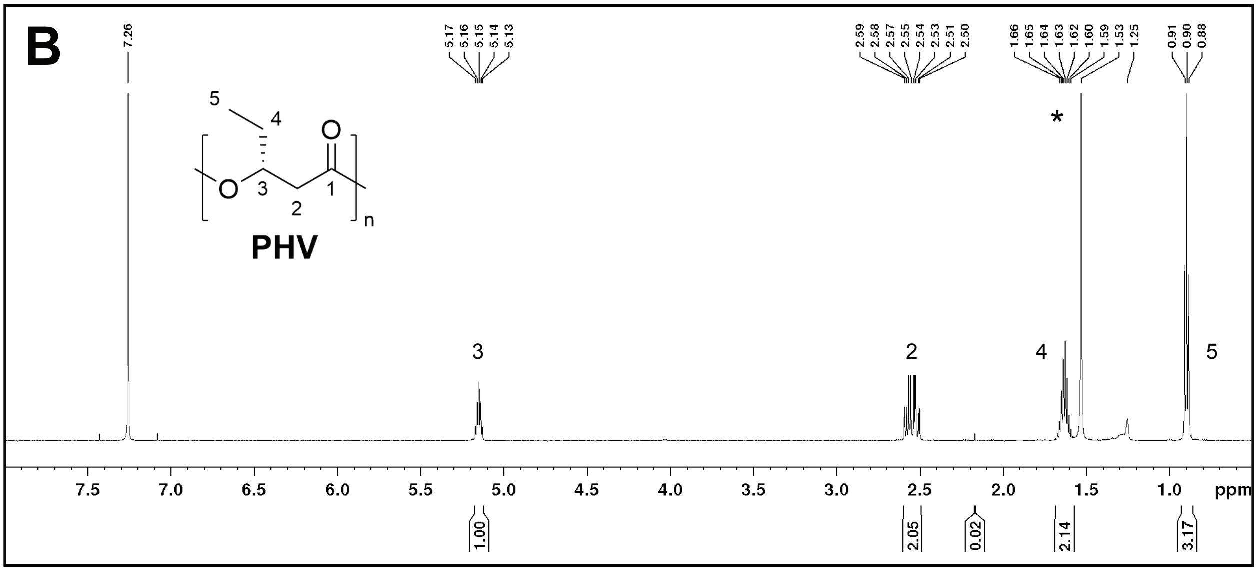

Figure S2-B ^{1}H-NMR (600 MHz, $CDCl_3$); δ 5.17-5.13 (p, 1H), 2.59-2.50 (m, 2H), 1.66-1.59 (m, 2H), 0.91-0.88 (t, 3H). The asterisk (*) at δ 1.53 denotes a water impurity (Fulmer et al. 2010).

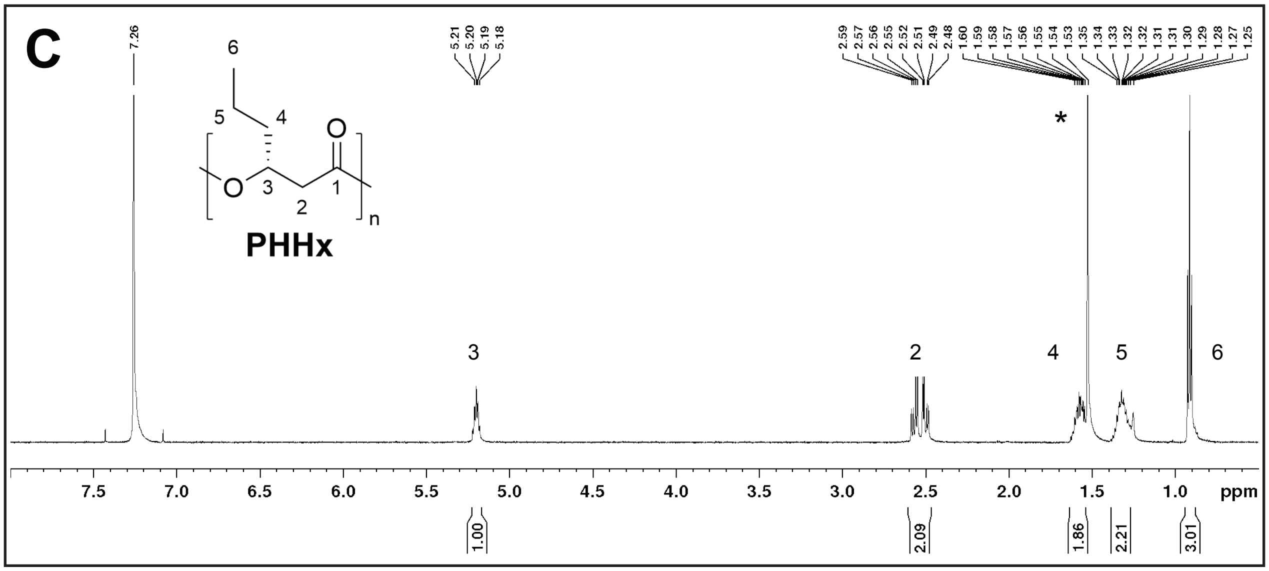

Figure S2-C ^{1}H-NMR (600 MHz, $CDCl_3$); δ 5.21-5.18 (p, 1H), 2.59-2.48 (m, 2H), 1.60-1.54 (m, 2H), 1.35-1.27 (m, 2H), 0.91 (t, 3H). The asterisk (*) at δ 1.53 denotes a water impurity (Fulmer et al. 2010).

Table S1: Poly(3-hydroxydecanoate) yield comparison between LSBJ, RSC02, RSC04, and RSC06

Strain	CDW[a] (g L^{-1})	PHA[a] (wt%)	PHA Concentration[a] (mg L^{-1})
LSBJ	1.13 ± 0.02	17.4 ± 3.7	196 ± 40
RSC02	1.33 ± 0.04*	26.4 ± 4.7*	353 ± 77*
RSC04	0.93 ± 0.00*	16.5 ± 1.9	154 ± 18
RSC06	0.99 ± 0.02*	17.9 ± 3.0	179 ± 31

[a] All values are averages of biological triplicate experiments plus or minus the standard deviation about those averages.

* Denotes statistically significant difference compared to LSBJ (Student's *t*-test, two-tailed, α = 0.05).

References

Fulmer GR, Miller AJM, Sherden NH, Gottlieb HE, Nudelman A, Stoltz BM, Bercaw JE, Gold berg KI (2010) NMR Chemical Shifts of Trace Impurities: Common Laboratory Solvents, Organics, and Gases in Deuterated Solvents Relevant to the Organometallic Chemist. Organometallics 29:2176–2179. doi: 10.1021/om100106e

Pinto A, Ciesla JH, Palucci A, Sutliff BP, Nomura CT (2016) Chemically Intractable No More: In Vivo Incorporation of "Click"-Ready Fatty Acids into Poly-[(R)-3-hydroxyalkanoates] in *Escherichia coli*. ACS Macro Lett 5:215–219. doi: 10.1021/acsmacrolett.5b00823
